# Trends and projections of the global and regional burden of multiple myeloma in adults aged 40 and over, 1990–2044

**DOI:** 10.1038/s41598-025-96981-w

**Published:** 2025-04-19

**Authors:** Xi Liu, Haihui Zhuang, Fenglin Li, Minli Shen, Ying Lu, Renzhi Pei

**Affiliations:** 1 Department of Hematology, The Affiliated People’s Hospital of Ningbo University, Ningbo, China; 2 Institute of Hematology, Ningbo University, Ningbo, China

**Keywords:** Multiple myeloma, Global burden of disease, Age structure analysis., Haematological cancer, Oncology

## Abstract

The objective is to examine the data from the global burden of disease (GBD) 2021 to report on the global, regional, and national trends and rates related to the incidence, prevalence, mortality, and disability-adjusted life years (DALYs) for multiple myeloma in adults aged 40 and above. Data from the GBD 2021 was used in this study to evaluate the rates of incidence, prevalence, mortality, and DALYs related to multiple myeloma across global, regional, and national scales. To analyze these trends, joinpoint regression was applied to calculate the average annual percentage changes (AAPC). Further, the analysis was divided according to age, gender, region, and socio-demographic index (SDI). Projections extending to 2044 were also generated using the Nordpred model. From 1990 to 2021, an upward trend has been observed globally in the incidence, prevalence, mortality, and DALYs of multiple myeloma among adults aged 40 and older, with average annual percentage changes (AAPCs) of 0.53 (95% CI 0.41–0.64), 1.2 (95% CI 1.07–1.33), 0.19 (95% CI 0.07–0.31), and 0.15 (95% CI 0.04–0.26), respectively. Notably, middle SDI countries exhibited the most accelerated disease progression, demonstrating a 2.34 (95% CI 2.19–2.48) annual increase in age-standardized incidence rate (ASIR)—over fourfold higher than the global average. The increasing global burden of multiple myeloma in adults aged 40 and older, especially in areas and nations with lower SDI levels, underscores the pressing requirement for customized public health initiatives and policies to tackle this escalating issue.

## Introduction

Multiple myeloma (MM) is defined as a malignant disease characterized by the abnormal growth of plasma cells in the bone marrow, leading to the production of monoclonal immunoglobulin or its fragments (M protein). This condition causes damage to affect organs or tissues, manifesting as symptoms such as bone damage, kidney dysfunction, elevated calcium levels, and anemia^1^. Recent reports estimate 176,404 new MM cases and 117,077 related deaths, representing 0.9% of all new cancer diagnoses and 1.2% of cancer-related deaths worldwide^2–4^. Research indicates a sharp rise in the incidence of multiple myeloma starting at age 40, with the highest rates observed in individuals aged 65 to 69. This data highlights a significant increase in the burden of MM with advancing age. Consequently, a detailed analysis of the global impact of MM is essential for informing health policies, resource distribution, research efforts, and patient management^5,6^. Beyond clinical outcomes, MM imposes substantial economic burdens and patients often face prolonged disability. These impacts are magnified in resource-limited settings where diagnostic delays and costly novel therapies exacerbate health inequities.

## Methods

### Overview

The GBD 2021 offers extensive data on MM at global, regional, and national levels, covering a wide age spectrum from 40 to over 95 years. This age range is divided into specific groups: 40–44, 45–49, 50–54, 55–59, 60–64, 65–69, 70–74, 75–79, 80–84, 85–89, 90–94, and 95+. The study evaluates the impact of MM using various indicators, such as the number of incidence cases, prevalence cases, mortality cases, as well as DALYs and their respective rates.

### Data sources

Data for the study was retrieved through the Global Health Data Exchange’s search function by setting specific parameters, including selecting “GBD Estimate” to identify causes of mortality or injury. Essential metrics such as “Incidence,” “Prevalence,” “Mortality,” and “DALYs” were focused on, specifically targeting “multiple myeloma” across various global regions and categorized by SDI levels. The search parameters also encompassed age groups from 40 to over 95 years, included both genders, and covered all available years to ensure a thorough analysis.

### Uncertainty intervals

To address uncertainties stemming from primary data sources, data processing, measurement inaccuracies, and model choices, the 2021 GBD study utilized ensemble and meta-regression models to generate 1000 simulations for each entity. The ultimate estimates were provided with 95% uncertainty intervals (UIs), which were calculated from the 25 and 975th percentiles of these 1000 simulations.

### Inequality analysis

We performed a decomposition analysis to evaluate how population growth, aging, and changes in epidemiology contribute to the disease burden across various SDI levels. The goal was to pinpoint the main factors affecting disease burden at different stages of socio-economic development.

To explore health disparities further, we computed the slope index and concentration indices for incidence, DALYs, prevalence, and mortality from 1990 to 2021. These indices reveal changes in disease burden over time, showing how inequalities in regional development might have impacted health outcomes^4,7^.

### Statistical analysis

Our research aimed to estimate global trends in the incidence, prevalence, mortality, and DALYs related to MM. We analyzed these trends by gender, age, and SDI, and examined patterns at both regional and national levels. Age-standardized rates, such as ASIR, age-standardized prevalence rate (ASPR), age-standardized mortality rate (ASMR), and age-standardized DALYs rate (ASDR), were calculated using the global standard population from the GBD Study 2021, with their 95% CIs reported. These rates were expressed per 100,000 people. Joinpoint regression analysis was employed to determine the AAPC and its 95% CIs for trend assessment. To forecast future trends, we used the Nordpred method, a well-regarded technique in academic circles, across SDI regions and 21 global regional forecasting models. This approach utilizes age-period-cohort analysis to project future disease patterns, providing a comprehensive framework for anticipating future health scenarios based on historical data and demographic changes. All statistical analyses were performed with R Studio software (version 4.3.2)^8,9^.

## Results

### The incidence, prevalence, mortality and dalys of MM at global level

Between 1990 and 2021, there has been a rise in the incidence, prevalence, mortality, and DALYs of MM among adults over 40. For a detailed breakdown, refer to (Table 1).

**Table 1:** The cases, rates and AAPC of MM incidence, prevalence, mortality, and dalys of MM in aged 40 years and older at the global level from 1990 to 2021.

	Incidence				AAPC (95% UI)	Prevalence				AAPC (95% UI)
	1990		2021			1990		2021		
	Cases (95% UI)	ASIR/100,000 (95% UI)	Cases (95% UI)	ASIR/100,000(95% UI)		Cases (95% UI)	ASPR/100,000 (95% UI)	Cases (95% UI)	ASPR/100,000 (95% UI)	
Global	54696.79 (59234.3–50319.53)	3.9 (4.22–3.59)	146218.04 (162921.37–126091.14)	5.03 (5.61–4.34)	0.53 (0.41–0.64)	121073.31 (130833.41–111890.72)	8.63 (9.33–7.98)	385765.23 (428005.61–336289.4)	13.28 (14.74–11.58)	1.2 (1.07–1.33)
Sex										
Male	27865.13 (30774.8–25380.52)	4.08 (4.5–3.71)	80943.09 (90857.52–68430.33)	5.73 (6.44–4.85)	0.71 (0.59–0.83)	61328.09 (67255.3–55983.27)	8.97 (9.84–8.19)	217891.78 (244656.64–184912.53)	15.44 (17.33–13.1)	1.5 (1.39–1.62)
Female	26831.66 (29914.29–24127.05)	3.73 (4.16–3.36)	65274.96 (76469.21–53221.01)	4.37 (5.12–3.56)	0.28 (0.15–0.41)	59745.22 (67035.16–53247.43)	8.31 (9.32–7.41)	167873.45 (195103.31–139016.16)	11.24 (13.07–9.31)	0.82 (0.61–1.04)
Age, years										
40–44	1087.254 (1218.485–969.8139)	0.3795196 (0.4253276 −0.3385257)	2531.024 (3034.347–2017.7071)	0.5059509 (0.6065651 −0.4033391)	0.94 (0.85–1.03)	3355.413 (3646.905–3071.081)	1.171249 (1.272998–1.071999)	8993.865 (10448.401–7374.421)	1.797871 (2.088632–1.474145)	1.38 (1.27–1.49)
45–49	1941.660 (2124.924–1773.515)	0.8362176 (0.9151446 −0.7638023)	4699.334 (5389.049–3981.168)	0.9924578 (1.1381197–0.8407876)	0.53 (0.35–0.71)	6005.617 (6490.707–5524.782)	2.586449 (2.795363–2.379367)	16816.499 (18863.901–14418.971)	3.551496 (3.983889–3.045159)	1.09 (0.89–1.3)
50–54	3613.493 (3952.964–3320.844)	1.699896 (1.859593–1.562225)	8655.090 (9842.527–7339.269)	1.945301 (2.212187–1.649559)	0.43 (0.31–0.56)	11134.89 (12065.80–10315.42)	5.238186 (5.676113–4.852683)	31360.24 (35024.33–27049.49)	7.048464 (7.871999–6.079589)	0.96 (0.78–1.15)
55–59	5353.516 (5918.793–4931.555)	2.890668 (3.195893–2.662827)	13165.833 (14917.583–11324.051)	3.326991 (3.769656–2.861574)	0.52 (0.13–0.9)	15128.70 (16526.79–14110.87)	8.168847 (8.923757–7.619261)	44468.03 (49395.81–38548.07)	11.237020 (12.482262–9.741053)	1.19 (1–1.37)
60–64	7873.022 (8554.165–7356.288)	4.901973 (5.326073–4.580240)	17742.637 (19856.836–15611.077)	5.543745 (6.204333–4.877732)	0.45 (0.28–0.62)	20296.91 (21810.06–18980.36)	12.63745 (13.57958–11.81773)	55099.63 (60464.95–49122.30)	17.21606 (18.89247–15.34842)	1.05 (0.79–1.31)
65–69	9088.569 (9791.899–8482.861)	7.352649 (7.921642–6.862631)	21839.434 (24295.730–18977.245)	7.917366 (8.807838–6.879748)	0.25 (0.14–0.37)	20811.51 (22372.42–19372.52)	16.83650 (18.09928–15.67236)	60684.41 (66677.33–53920.98)	21.99969 (24.17228–19.54777)	0.97 (0.67–1.28)
70–74	8268.212 (8918.250–7765.368)	9.766241 (10.53405–9.172292)	23966.815 (26374.103–21447.068)	11.643464 (12.81296–10.419330)	0.66 (0.47–0.86)	16969.20 (18223.57–15906.30)	20.04367 (21.52531–18.78819)	63305.20 (69469.90–57457.40)	30.75468 (33.74960–27.91373)	1.4 (1.24–1.57)
75–79	8163.618 (8691.768–7671.393)	13.26220 (14.12021–12.46256)	20438.762 (22354.768–18261.399)	15.49748 (16.95027–13.84652)	0.53 (0.42–0.65)	14753.82 (15852.37–13712.83)	23.96832 (25.75296–22.27718)	47981.24 (52771.15–42194.69)	36.38128 (40.01319–31.99369)	1.36 (1.17–1.56)
80–84	5634.716 (6033.064–4955.337)	15.92811 (17.05415–14.00765)	16664.289 (18241.435–13914.347)	19.02684 (20.82758–15.88703)	0.68 (0.4–0.96)	8604.368 (9403.585–7552.312)	24.32266 (26.58188–21.34873)	33566.326 (37770.014–27742.237)	38.32514 (43.12480–31.67535)	1.66 (1.34–1.99)
85–89	2730.656 (2984.102–2336.910)	18.07056 (19.74778–15.46488)	10772.900 (12075.169–8786.929)	23.56185 (26.41010–19.21825)	0.94 (0.55–1.33)	3221.814 (3553.519–2715.210)	21.32088 (23.51599–17.96835)	17190.835 (19660.097–13690.952)	37.59878 (42.99940–29.94404)	2.09 (1.63–2.55)
90–94	801.3182 (887.6238–647.8645)	18.69972 (20.71377–15.11870)	4699.3912 (5341.7909–3674.0291)	26.26918 (29.86013–20.53749)	1.18 (0.96–1.4)	718.0322 (805.5638–573.1196)	16.75615 (18.79880–13.37443)	5746.1158 (6823.7322–4369.0087)	32.12027 (38.14405–24.42237)	2.24 (1.96–2.52)
95 plus	140.7567 (158.2631–107.7858)	13.82561 (15.54516–10.58710)	1042.5361 (1198.0297–756.8548)	19.12802 (21.98095–13.88646)	1 (0.52–1.48)	73.03772 (82.12119–55.91730)	7.174019 (8.066230–5.492391)	552.83676 (635.98774–400.86949)	10.143219 (11.668839–7.354987)	1.08 (0.6–1.56)
SDI										
High SDI	33039.81 (34786.76–30837.6)	8.81 (9.28–8.22)	67859.98 (73703.17–59660.32)	9.31 (10.09–8.27)	0.16 (−0.01–0.33)	75475.62 (81495.76–69453.71)	20.44 (22.07–18.81)	194004.88 (214720.98–170898.28)	34.14 (37.79–30.07)	1.02 (0.83–1.21)
High-midlle SDI	12291.08 (13496.35–11255.32)	3.74 (4.11–3.41)	34130.32 (38782.1–28569.77)	5.39 (6.13–4.51)	1.04 (0.95–1.13)	29674.46 (33816.18–26109.93)	8.73 (9.96–7.66)	98025.51 (113151.85–80843.64)	14.59 (16.86–12.01)	1.71 (1.46–1.95)
Middle SDI	4993.74 (6398.8–4254.06)	1.49 (1.9–1.27)	27639.71 (33122.99–21798.43)	3.03 (3.64–2.39)	2.34 (2.19–2.48)	9092.58 (11528.22–7801.52)	2.55 (3.22–2.18)	64174.13 (76839.85–51212.67)	6.8 (8.15–5.43)	3.23 (3.09–3.37)
Low-middle SDI	3099.02 (4138.3–2174.29)	1.57 (2.1–1.1)	12770.03 (17867.2–10622.53)	2.7 (3.79–2.24)	1.8 (1.7–1.9)	4875.48 (6466.16–3445)	2.32 (3.08–1.64)	23027.53 (31966.3–19225.86)	4.62 (6.43–3.86)	2.28 (2.16–2.39)
Low SDI	1198.44 (1710.31–654.68)	1.65 (2.35–0.91)	3639.71 (4973.64–2411.57)	2.24 (3.05–1.49)	1 (0.87–1.12)	1788.58 (2543.66–971.51)	2.29 (3.26–1.26)	6045.19 (8320.53–3991.78)	3.44 (4.73–2.29)	1.33 (1.19–1.47)

	Mortality				AAPC (95% UI)	DALYs				AAPC (95% UI)
	1990		2021			1990		2021		
	Cases (95% UI)	ASMR/100,000 (95% UI)	Cases (95% UI)	ASMR/100,000 (95% UI)		Cases (95% UI)	ASDR/100,000 (95% UI)	ASDR/100,000 (95% UI)	rates/100,000 (95% UI)	
Global	46841.96 (50977.53–42876.96)	3.34 (3.63–3.06)	114780.5 (128949.18–98163.83)	3.95 (4.44–3.38)	0.19 (0.07–0.31)	1080715.28 (1182779.75–991750.61)	77.05 (84.32–70.7)	2504670.78 (2837449.96–2145369.63)	86.23 (97.69–73.86)	0.15 (0.04–0.26)
Sex										
Male	23649.01 (26420.33–21291.23)	3.46 (3.86–3.11)	62178.59 (70542.93–52418.75)	4.41 (5–3.71)	0.32 (0.19–0.46)	567679.69 (638603.44–508747.72)	83.02 (93.39–74.4)	1389987.51 (1599448.4–1156178.52)	98.47 (113.31–81.91)	0.28 (0.16–0.4)
Female	23192.95 (26024.38–20770.52)	3.23 (3.62–2.89)	52601.91 (61846.44–42373.33)	3.52 (4.14–2.84)	–0.01 (−0.11–0.09)	513035.58 (582733.7–462214.88)	71.37 (81.06–64.3)	1114683.27 (1324734.7–897609.1)	74.65 (88.72–60.12)	−0.02 (−0.13–0.08)
Age, years										
40–44	752.7364 (857.8332–659.1167)	0.2627521 (0.2994374 − 0.2300729)	67859.98 (73703.17–59660.32)	9.31 (10.09–8.27)	0.57 (0.47–0.68)	36604.3 (41622.12–32017.91)	12.77719 (14.52872–11.17625)	76329.57 (92735.09–60150.41)	15.25826 (18.53772–12.02405)	0.58 (0.48–0.69)
45–49	1342.150 (1500.928–1196.747)	0.5780260 (0.6464070 − 0.5154051)	2886.263 (3391.458–2409.797)	0.6095533 (0.7162460 − 0.5089279)	0.21 (0.11–0.31)	58646.29 (65500.95–52336.56)	20.44 (22.07–18.81)	126771.22 (148050.78-105855.05)	26.77296 (31.26702–22.35565)	0.22 (0.12–0.32)
50–54	2494.207 (2791.246–2260.565)	1.173350 (1.313086–1.0634375)	5245.479 (6091.336–4362.693)	1.178963 (1.369077–0.9805502)	0.03 (−0.17–0.22)	75475.62 (81495.76–69453.71)	45.74656 (51.34413–41.49675)	205705.94 (239598.5–171559.34)	34.14 (37.79–30.07)	1.02 (0.83–1.21)
55–59	3929.540 (4356.315–3588.879)	2.121782 (2.352223–1.937840)	8522.473 (9833.737–7193.496)	2.153619 (2.484974–1.817788)	0.09 (−0.09–0.27)	134382.6 (148595.8–122445.7)	72.56079 (80.23532–66.11543)	293696.3 (338177.4–247461.0)	74.21671 (85.45704–62.53311)	0.11 (−0.06–0.29)
60–64	6068.965 (6633.028–5621.051)	3.778715 (4.129917–3.499831)	17742.637 (19856.836–15611.077)	5.543745 (6.204333–4.877732)	0.06 (−0.04–0.15)	179158.9 (195841.4–165895.9)	111.5495 (121.9366–103.29161)	361982.7 (411945.3–313968.5)	113.1027 (128.7137–98.10050)	0.08 (−0.01–0.17)
65–69	7435.240 (8077.130–6947.408)	6.015106 (6.534395–5.620450)	16057.317 (18306.202–13821.841)	5.821198 (6.636477–5.010779)	−0.06 (−0.15–0.03)	75475.62 (81495.76–69453.71)	20.44 (22.07–18.81)	403034.1 (459461.0–347759.1)	34.14 (37.79–30.07)	−0.08 (−0.21–0.04)
70–74	7288.502 (7963.561–6821.414)	8.609028 (9.406394–8.057313)	18717.228 (20750.327–16529.546)	9.093130 (10.080842–8.030319)	0.23 (0.11–0.36)	149676.4 (163293.2–140288.7)	176.7947 (192.8785–165.7061)	387872.1 (430964.3–342765.6)	188.4345 (209.3694–166.5210)	0.26 (0.14–0.39)
75–79	7586.193 (8062.238–7110.419)	12.32415 (13.09751–11.55123)	16999.484 (18616.534–15154.236)	12.88968 (14.11580–11.49054)	0.15 (0.06–0.25)	124553.5 (132349.7–116646.1)	202.3433 (215.0087–189.4973)	282146.9 (309892.9–250884.2)	213.9350 (234.9732–190.2304)	0.19 (0.1–0.28)
80–84	5712.896 (6112.884–5095.232)	16.14911 (17.27978–14.40311)	15265.414 (16778.086–12975.921)	17.42964 (19.15677–14.81556)	0.33 (0.07–0.58)	73724.53 (78940.15–65899.99)	208.4031 (223.1465–186.2848)	198557.23 (217840.03–169145.48)	226.7074 (248.7239–193.1258)	0.35 (0.09–0.6)
85–89	3026.929 (3287.719–2604.554)	20.03120 (21.75702–17.23607)	10683.712 (11878.398–8792.691)	23.36678 (25.97973–19.23085)	0.57 (0.34–0.8)	30972.13 (33664.25–26656.34)	204.9632 (222.7787–176.4027)	110110.77 (122524.50–90726.51)	240.8278 (267.9783–198.4317)	0.59 (0.36–0.81)
90–94	985.0879 (1088.110–803.5306)	22.98821 (25.39235–18.75135)	5094.9203 (5736.339–3961.1079)	28.48015 (32.06563–22.14224)	0.76 (0.53–1)	8722.469 (9640.959–7102.293)	203.5493 (224.9834–165.7405)	45561.784 (51416.672–35667.701)	254.6863 (287.4146–199.3793)	0.8 (0.56–1.03)
95 plus	219.5133 (246.5417–168.0408)	21.56136 (24.21618–16.50555)	1577.1396 (1812.7187–1149.1688)	28.93670 (33.25901–21.08447)	0.9 (0.37–1.45)	1815.132 (2044.374–1394.025)	178.2886 (200.8055–136.9260)	12902.105 (14843.396–9426.697)	236.7225 (272.3405–172.9571)	0.97 (0.61–1.32)
SDI										
High SDI	27948.72 (29101.84–26153.15)	7.41 (7.72–6.93)	51228.16 (54948.17–45009.74)	6.74 (7.2–5.99)	−0.31 (−0.44–0.18)	598805.37 (620763.46–569393.89)	162.06 (167.98–154.23)	965116.61 (1027518.37–873702.23)	169.83 (180.82–153.75)	−0.52 (−0.63–0.41)
High-midlle SDI	9945.09 (10893.48–9212.86)	3.1 (3.4–2.85)	25098.61 (28267.93–21098.23)	3.76 (4.24–3.16)	0.66 (0.56–0.77)	243076.79 (266291.05–226564.35)	72.01 (78.95–66.89)	562816.18 (636541.17–472496.21)	83.94 (95.01–70.39)	0.5 (0.37–0.62)
Middle SDI	4633.92 (5984.68–3935.51)	1.44 (1.84–1.21)	22840.8 (27361.06–17941.27)	2.57 (3.07–2.01)	1.9 (1.75–2.06)	124016.95 (160020.25-105269.71)	34.56 (44.52–29.31)	576551.45 (689747.99–452704.89)	59.56 (71.26–46.73)	1.89 (1.77–2.01)
Low-middle SDI	3054.03 (4087.47–2140.72)	1.6 (2.15–1.13)	11956.56 (16806.33–9903.95)	2.6 (3.66–2.15)	1.58 (1.44–1.71)	81192.9 (108356.22–56651.92)	38.51 (51.44–26.95)	304729.75 (425282.3–252623.82)	61.51 (86.04–50.97)	1.54 (1.44–1.63)
Low SDI	1197.14 (1712.36–657.65)	1.7 (2.44–0.95)	3520.08 (4813.93–2336.33)	2.26 (3.08–1.51)	0.94 (0.85–1.03)	32122.89 (45913.69–17358.86)	40.99 (58.65–22.42)	92470.56 (126925.94–60829.13)	52.84 (72.34–34.96)	0.82 (0.73–0.91)

### Global trends

Globally, the incidence of new MM cases in adults over 40 rose from 54,696.79 (95% CI 59,234.3 to 50,319.53) in 1990 to 146,218.04 (95% CI 162,921.37 to 126,091.14) in 2021. In 2021, the ASIR of MM was 5.03 per 100,000 (95% CI 5.61 to 4.34), up from 3.9 per 100,000 in 1990 (95% CI 4.22 to 3.59), reflecting an AAPC of 0.53 per 100,000 (95% CI 0.41 to 0.64). The ASPR increased from 8.63 per 100,000 (95% CI 9.33 to 7.98) in 1990 to 13.28 per 100,000 (95% CI 14.74 to 11.58) in 2021, with an AAPC of 1.2 (95% CI 1.07 to 1.33). The ASMR and ASDR exhibited a gradual increase, with AAPCs of 0.19 (95% CI 0.07 to 0.31) and 0.15 (95% CI 0.04 to 0.26), respectively (Table 1).

### Global trends by sex and age groups

From 1990 to 2021, the global ASIR for MM increased in both males and females aged 40 and over. For males, the ASIR rose from 4.08 per 100,000 (95% CI 4.5 to 3.71) in 1990 to 5.73 per 100,000 (95% CI 6.44 to 4.85) in 2021, with an AAPC of 0.71 (95% CI 0.59 to 0.83). For females, the ASIR increased from 3.73 per 100,000 (95% CI 4.16 to 3.36) in 1990 to 4.37 per 100,000 (95% CI 5.12 to 3.56) in 2021, with an AAPC of 0.28 (95% CI 0.15 to 0.41). Generally, the rate of increase in incidence was higher for males compared to females. The ASIR for MM grew across all twelve age groups from 40 years and older. The most notable rise was observed in the 90–94 years age group, where the ASIR increased from 18.70 per 100,000 (95% CI 20.71 to 15.12) in 1990 to 26.27 per 100,000 (95% CI 29.86 to 20.54) in 2021, with an AAPC of 1.18 (95% CI 0.96 to 1.4) (Table 1).

### Global trends by SDI

From 1999 to 2021, the ASIR for MM increased across all SDI quintiles. The most notable rise was observed in the middle SDI quintile, where the ASIR grew from 1.49 per 100,000 (95% CI 1.9 to 1.27) in 1990 to 3.03 per 100,000 (95% CI 3.64 to 2.39) in 2021, with an AAPC of 2.34 (95% CI 2.19 to 2.48) (Table 2). In 2021, the global rates per 100,000 for ASIR, ASPR, ASMR, and ASDR were 5.03 (95% CI 5.61 to 4.34), 13.28 (95% CI 14.74 to 11.58), 3.95 (95% CI 4.44 to 3.38), and 86.23 (95% CI 97.69 to 73.86), respectively (Table 1).

**Table 2:** The rates, AAPC of MM incidence, prevalence, mortality, and dalys of MM at the regional levels from 1990 to 2021.

Location	Incidence	1990	2021	AAPC (95% UI)	Prevalence	1990	2021	AAPC (95%UI)	Mortality	1990	2021	AAPC (95%UI)	DALYs	1990	2021	AAPC (95%UI)
		ASIR/100,000 (95% UI)	ASIR/100,000 (95% UI)			ASPR/100,000 (95% UI)	ASPR/100,000 (95%UI)			ASMR/100,000 (95%UI)	ASMR/100,000 (95%UI)			ASDR/100,000 (95%UI)	ASDR/100,000 (95%UI)	
Central Latin America		3.2 (3.39–3.01)	4.76 (5.39–4.17)	1.31 (0.85–1.78)		5.73 (6.2–5.32)	11.26 (13.13–9.67)	2.24 (1.81–2.68)		3.01 (3.17–2.85)	3.89 (4.38–3.43)	0.88 (0.42–1.34)		75.18 (78.88–71.47)	98.42 (111.09–87.07)	0.88 (0.59–1.18)
High-income Asia Pacific		5.9 (6.7–5.21)	5.7 (6.93–4.47)	−0.17 (−0.27–0.07)		14.76 (17.92–12.08)	18.45 (24.13–13.44)	0.7 (0.44–0.96)		4.57 (4.87–4.18)	3.8 (4.28–3.13)	-0.67 (-0.79–0.54)		99.38 (105.72–92.15)	74.98 (84.67–63.39)	−0.97 (−1.1–0.85)
Oceania		0.94 (1.47–0.56)	1.02 (1.52–0.58)	0.28 (0.16–0.4)		1.45 (2.24–0.88)	1.67 (2.46–0.96)	0.48 (0.29–0.68)		0.94 (1.48–0.55)	1 (1.5–0.56)	0.19 (0.04–0.35)		21.62 (33.9-12.75)	23.36 (35.21–12.96)	0.27 (0.11–0.43)
Southeast Asia		0.87 (1.42–0.66)	1.55 (2.56–1.16)	1.87 (1.72–2.02)		1.44 (2.35–1.1)	3.16 (5.21–2.33)	2.55 (2.43–2.67)		0.84 (1.38–0.64)	1.36 (2.27–1.03)	1.56 (1.43–1.7)		20.72 (33.97–15.9)	33.13 (54.57–25.14)	1.52 (1.42–1.62)
Australasia		12.3 (14.18–10.61)	16.21 (20.11–12.47)	0.99 (0.36–1.62)		37.89 (45.86–31.07)	68.32 (88.47–50.49)	2.05 (1.7–2.4)		8.38 (9.26–7.48)	8.58 (10.02–7.08)	0.17 (-0.62-0.95)		186.45 (206.43-167.03)	178.51 (207.94-149.65)	−0.07 (−0.76–0.64)
North Africa and Middle East		2.4 (3.45–1.61)	3.78 (5.2–2.72)	1.48 (1.39–1.57)		3.89 (5.58–2.6)	8.38 (11.55–5.9)	2.51 (2.39–2.64)		2.37 (3.41–1.59)	3.23 (4.43–2.34)	1.01 (0.91–1.12)		56.3 (80.86–37.59)	74.47 (102.01–53.85)	0.9 (0.82–0.99)
Western Europe		10.45 (11.32–9.59)	12.7 (14.07–11.1)	0.62 (0.41–0.84)		29.83 (33.19–26.78)	46.95 (53.32–41)	1.46 (1.26–1.65)		7.63 (8.03–7.1)	7.69 (8.32–6.79)	0.05 (−0.08–0.18)		167.43 (175.79-158.05)	157.17 (168.73–142.45)	−0.19 (−0.32–0.06)
East Asia		0.61 (1.19–0.42)	2.31 (3.04–1.48)	4.44 (3.89–5)		1.04 (1.96 − 0.74)	6.12 (8.25–3.86)	5.97 (5.5–6.44)		0.59 (1.17–0.4)	1.77 (2.34–1.14)	3.7 (2.54–4.87)		14.44 (28.47–10.02)	43.06 (57.09–27.46)	3.64 (3.16–4.12)
Central Europe		4.22 (4.54–3.89)	6.53 (7.27–5.81)	1.49 (1.33–1.65)		7.55 (8.29–6.85)	14.18 (16.38–12.28)	2.07 (1.82–2.33)		3.97 (4.25–3.68)	5.7 (6.23–5.11)	1.17 (0.97–1.38)		96.42 (102.48–89.52)	128.21 (139.94-115.56)	0.92 (0.7–1.14)
Southern Sub-Saharan Africa		4.3 (5.77–2.85)	6.7 (8.33–4.32)	1.43 (1.19–1.66)		6.85 (9.25–4.52)	11.48 (14.49–7.33)	1.66 (1.43–1.89)		4.27 (5.73–2.82)	6.38 (7.95–4.12)	1.28 (1.06–1.51)		105.31 (140.69–69.84)	158.63 (198.24-102.66)	1.31 (1.05–1.56)
Central Asia		0.76 (0.86 − 0.65)	1.4 (1.58–1.23)	1.88 (1.12–2.65)		1.31 (1.51–1.12)	2.63 (3.05–2.26)	2.26 (1.58–2.95)		0.72 (0.82 − 0.62)	1.28 (1.44–1.13)	1.95 (0.83–3.08)		20.13 (22.78–17.36)	34.24 (38.78–30.17)	1.76 (0.89–2.64)
Central Sub-Saharan Africa		1.07 (1.53–0.7)	1.28 (1.94 − 0.68)	0.59 (0.47–0.72)		1.48 (2.11–0.96)	1.96 (2.98–1.03)	0.88 (0.77-1)		1.11 (1.59–0.72)	1.29 (1.95 − 0.67)	0.48 (0.32–0.64)		27.13 (38.55–17.63)	31.53 (47.6-16.55)	0.49 (0.33–0.64)
Eastern Europe		3 (3.23–2.78)	5.14 (5.63–4.67)	1.74 (1.19–2.29)		6.47 (7.14–5.92)	13.74 (15.29–12.34)	2.42 (1.92–2.94)		2.52 (2.69–2.36)	3.85 (4.21–3.51)	1.46 (1.15–1.78)		69.25 (73.92–64.85)	99.42 (108.98–90.67)	1.3 (0.86–1.73)
Tropical Latin America		3.8 (4.06–3.52)	6.14 (6.7–5.53)	1.66 (1.22–2.09)		6.71 (7.31–6.13)	13.39 (15.03–11.89)	2.31 (1.96–2.67)		3.59 (3.81–3.33)	5.28 (5.69–4.78)	1.37 (0.89–1.85)		89.95 (95.18–84.29)	125.09 (133.45-115.12)	1.12 (0.66–1.57)
Western Sub-Saharan Africa		0.76 (1.08–0.38)	1.4 (2.08–0.57)	1.95 (1.84–2.06)		1.05 (1.5–0.53)	2.15 (3.31–0.84)	2.34 (2.21–2.47)		0.8 (1.14–0.4)	1.42 (2.06–0.59)	1.85 (1.73–1.98)		18.01 (25.49–9.08)	32.11 (47.52–12.94)	1.87 (1.76–1.99)
Eastern Sub-Saharan Africa		2.61 (3.8–1.42)	3.61 (5.03–2.2)	1.08 (1.05–1.1)		3.57 (5.22–1.92)	5.5 (7.65–3.33)	1.41 (1.34–1.47)		2.71 (3.96–1.49)	3.65 (5.09–2.24)	0.99 (0.96–1.01)		64.4 (94.43–34.58)	86.27 (121.27–51.97)	0.95 (0.93–0.98)
Andean Latin America		3.36 (4.54–2.39)	5.27 (7.3–3.87)	1.53 (0.9–2.16)		5.43 (7.37–3.83)	11.92 (17.1–8.36)	2.62 (2.02–3.23)		3.32 (4.47–2.36)	4.45 (6.06–3.3)	1 (0.37–1.63)		79.82 (107.42–56.91)	106.86 (144.71–79.13)	1.04 (0.24–1.85)
Caribbean		7.02 (8.28–6.03)	9.36 (11.25–7.67)	0.92 (0.31–1.53)		17.91 (22.78–14.35)	30.87 (38.7–24.14)	1.74 (1.07–2.42)		5.39 (6.18–4.78)	6.07 (7.09–5.12)	0.33 (−0.17–0.83)		124.44 (143.06–110.87)	145.32 (169.93-122.46)	0.48 (−0.02–0.99)
Southern Latin America		6.52 (7.51–5.65)	6.85 (7.94–5.87)	0.19 (−0.06–0.45)		11.63 (13.79–9.81)	16.57 (20.31–13.4)	1.13 (0.71–1.55)		6.15 (7.01–5.37)	5.59 (6.29–4.89)	−0.28 (−0.53–0.03)		143.55 (162.76–126.09)	127.82 (143.08-112.98)	−0.33 (−0.57–0.09)
South Asia		1.84 (2.39–1.13)	3.17 (4.35–2.4)	1.77 (1.57–1.96)		2.64 (3.42–1.61)	5.45 (7.39–4.09)	2.37 (2.21–2.52)		1.89 (2.46–1.17)	3.06 (4.22–2.33)	1.56 (1.37–1.74)		45.29 (58.72–27.73)	71.53 (98.23–54.48)	1.52 (1.38–1.66)
High-income North America		9.88 (10.4–9.2)	9.16 (9.85–8.24)	−0.27 (−0.45–0.09)		17.22 (18.84–15.72)	19.03 (21.4-16.76)	0.31 (0.17–0.46)		9.65 (10.04-9)	8.34 (8.86–7.49)	−0.49 (−0.74–0.25)		215.46 (222.91–204.78)	167.89 (176.82–154.8)	−0.84 (−1.01–0.67)

### Disease burden in the country

**Fig. 1:**
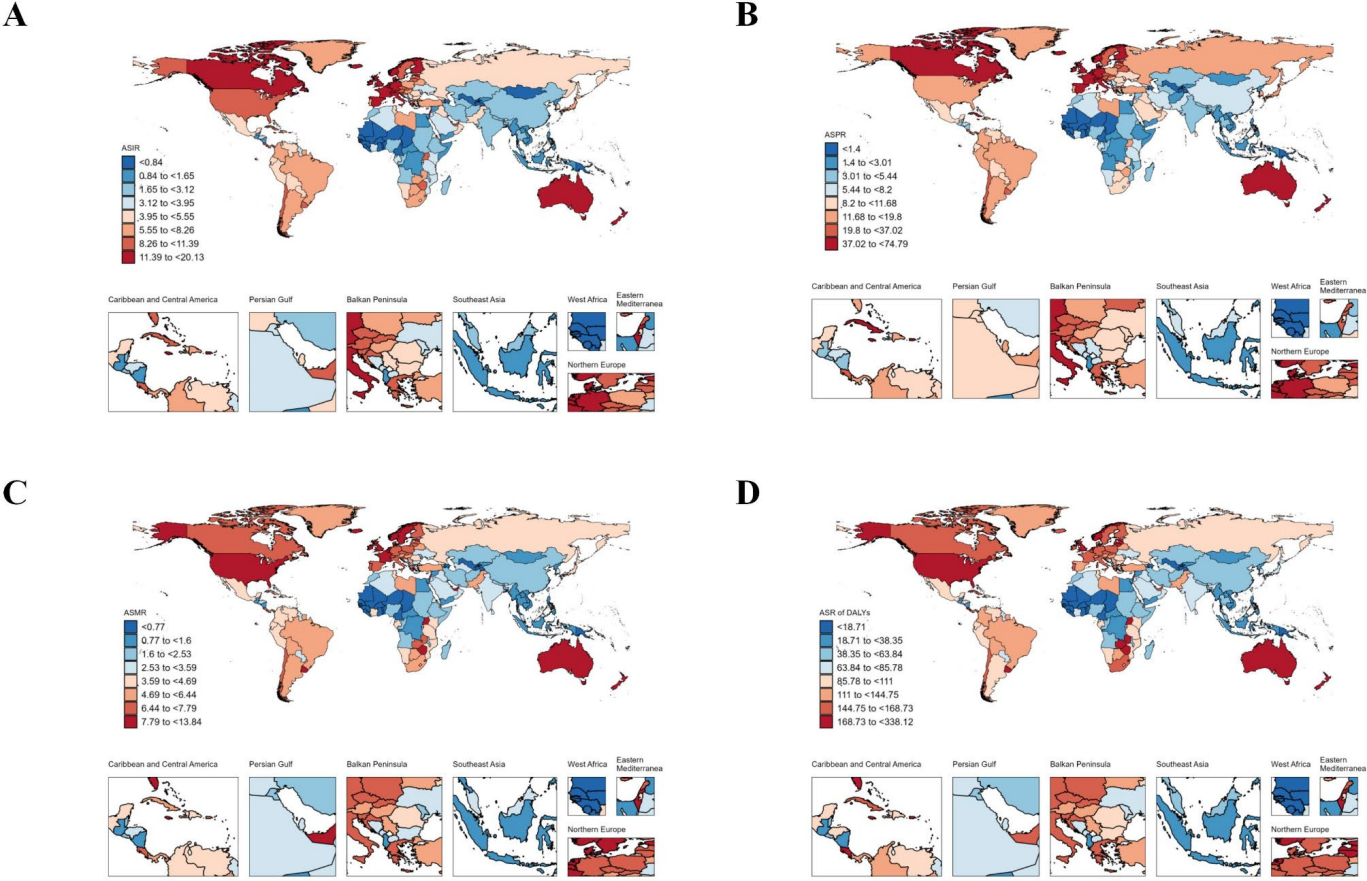
Geographical distribution of ASIR (**A**), ASPR (**B**), ASMR (**C)** and ASDR (**D**).

In 2021, the lowest ASIR were found in countries from Oceania, Central Sub-Saharan Africa, Western Sub-Saharan Africa, Central Asia, Southeast Asia, East Asia, and South Asia. Conversely, the highest ASIRs were observed in Western Europe and Australasia. The regions with the lowest ASPR included Oceania, Central Sub-Saharan Africa, Western Sub-Saharan Africa, Central Asia, Southeast Asia, South Asia, Eastern Sub-Saharan Africa, and East Asia, while Western Europe and Australasia had the highest ASPRs. For ASMR, the lowest rates were seen in Oceania, Central Asia, Central Sub-Saharan Africa, Southeast Asia, Western Sub-Saharan Africa, East Asia, South Asia, and North Africa and the Middle East. In contrast, Western Europe, High-income North America, and Australasia reported the highest ASMRs. Regarding ASDR, the lowest values were found in Oceania, Central Sub-Saharan Africa, Western Sub-Saharan Africa, Southeast Asia, Central Asia, East Asia, South Asia, North Africa and the Middle East, and High-income Asia Pacific. The highest ASDR were reported in the Caribbean, Western Europe, Southern Sub-Saharan Africa, High-income North America, and Australasia. (Table 2; Fig. 1A–D).

### Time trends of MM incidence in global and China

**Fig. 2:**
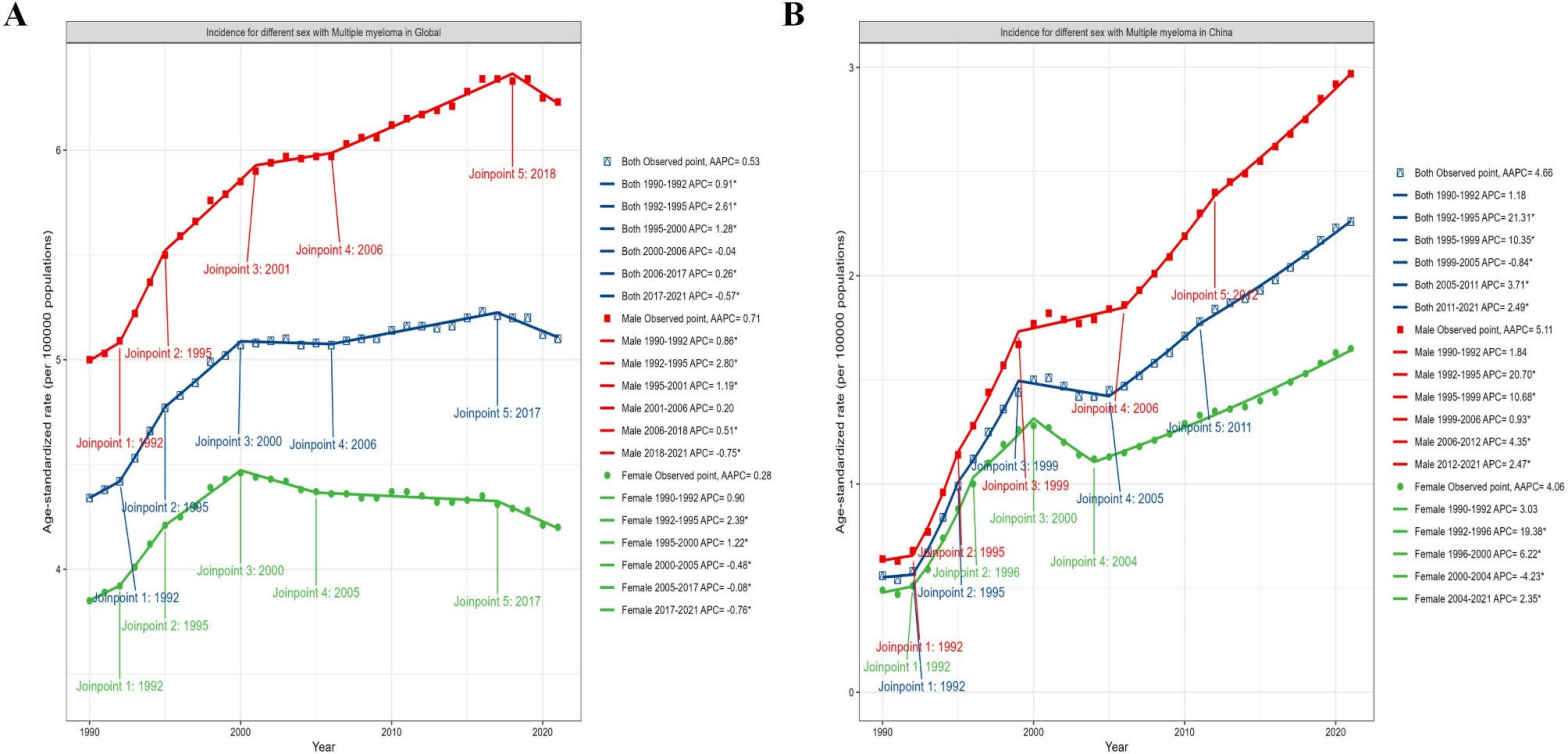
Trends in ASIR of MM in global (**A**), China (**B**).

Analysis of time trends for MM incidence data from the GBD 2021, covering the period from 1990 to 2021, revealed that the ASIR for men globally rose from 4.08 per 100,000 (95% CI 4.5 to 3.71) in 1990 to 5.73 per 100,000 (95% CI 6.44 to 4.85) in 2021, with an AAPC of 0.71 (95% CI 0.59 to 0.83). In contrast, Chinese men experienced a much more pronounced increase with an AAPC of 5.11 (95% CI 4.43 to 5.8). For females globally, the ASIR rose from 3.73 per 100,000 (95% CI 4.16 to 3.36) in 1990 to 4.37 per 100,000 (95% CI 5.12 to 3.56) in 2021, reflecting an AAPC of 0.28 (95% CI 0.15 to 0.41). This increase was tempered by a decline observed from 2000 to 2021. Conversely, the ASIR for MM among Chinese females demonstrated a rising trend with an AAPC of 4.06 (95% CI 3.39 to 4.73), despite an initial decrease from 2000 to 2004. (Supplemental Table 1; Fig. 2A, B).

### Decomposition analysis of change in incidence, prevalence, mortality, and dalys

**Fig. 3:**
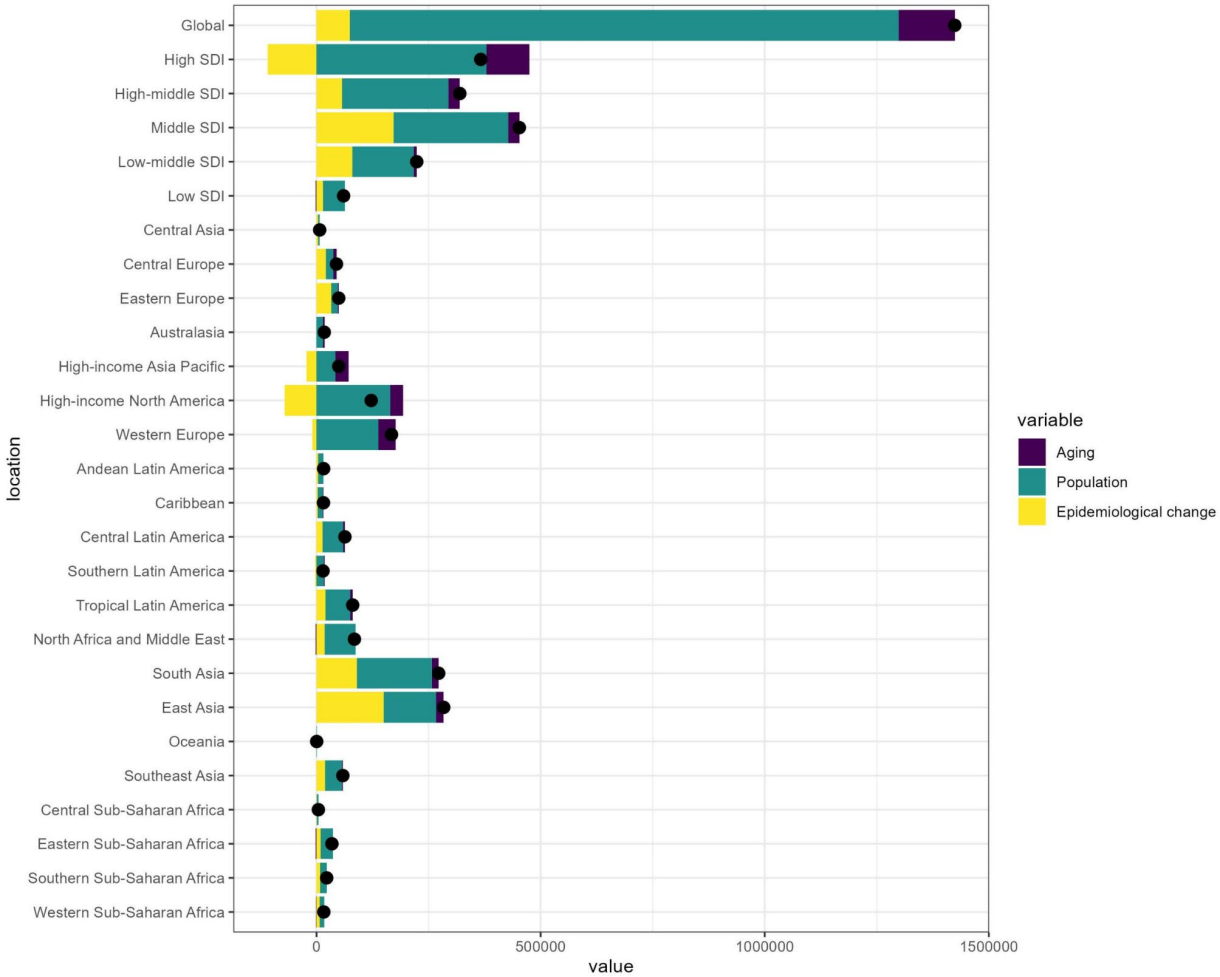
Changes in MM DALYs according to population-level determinants of population growth, aging, and epidemiological change from 1990 to 2021 at the global level and by different location.

To gain a clearer understanding of how population growth, aging, and epidemiological changes impact MM both globally and within various SDI regions, we performed a decomposition analysis. This analysis considered the impacts on DALYs (Fig. 3), incidence (Supplemental Fig. 1), prevalence (Supplemental Fig. 2), and mortality (Supplemental Fig. 3). The findings revealed that population growth was the predominant factor influencing both global and regional trends at various SDI levels. On a global scale, the changes in DALYs were primarily driven by population growth, accounting for 86.02% of the change, followed by aging at 8.76%, and epidemiological factors at 5.22%.In the SDI regions, the impact of population aging varied: high SDI contributed 26.25%, high-mid SDI 8.01%, middle SDI 5.41%, low-mid SDI 2.94%, and low SDI showed a negative contribution of −4.21%. Regarding population growth, its contributions were as follows: high SDI 103.5%, high-mid SDI 74.14%, middle SDI 56.6%, low-middle SDI 61.44%, and low SDI 80.56%. For epidemiological changes, the contributions were: high SDI −29.75%, high-mid SDI 17.85%, middle SDI 37.99%, low-middle SDI 35.61%, and low SDI 23.65%. (Supplemental Table 2).

### Slope indices and concentration indices

**Fig. 4:**
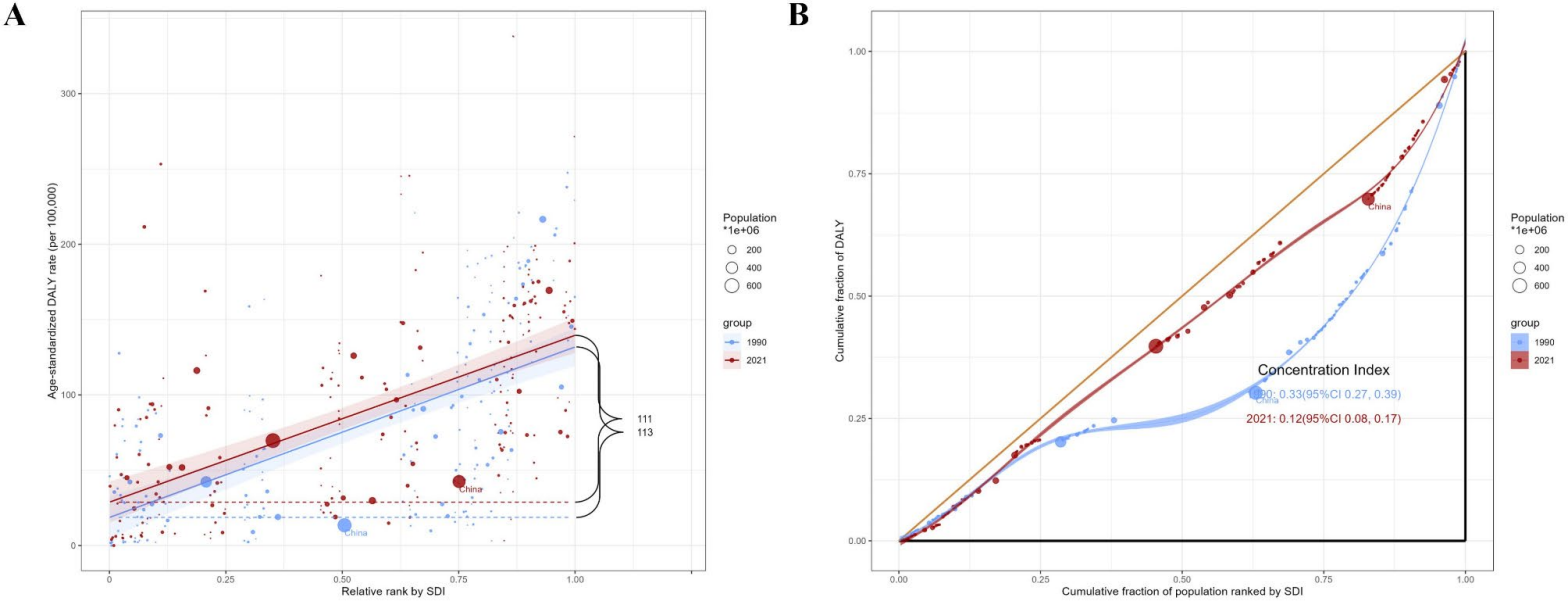
The health inequality indices for ASDR of MM in 1990 and 2021 based on the SDI of 204 countries and territories globally. Slope indices (the numbers adjacent to the brackets indicate the slopes) (**A**), Concentration indices. Each country or region is represented by a solid dot, with larger dots indicating a higher population. China highlighted for comparative purposes (**B**).

The Slope indices and Concentration indices inequality for ASDR of MM in 1990 and 2021are presented in (Supplemental Table 3). Positive slope values indicate a gradual increase in disease burden with increasing SDI. The slope for DALYs (Fig. 4A) in 1990 and 2021 were 113.19(95% CI 136.08–90.29), 110.76 (95% CI 130.77–90.75), respectively. The slopes of Incidence, prevalence and mortality all gradually increased from 1990 to 2021, from 6.37(95% CI 7.65–5.09), 14.02(95% CI 16.78–11.27), 5.07(95% CI 6.1–4.04), to 8.22(95% CI 9.57–6.87), 25.16(95% CI 29.36–20.97), 5.24(95% CI 6.14–4.34), respectively. ASDR of MM with concentration indices changed from 0.33(95% CI 0.27–0.39) to 0.12(95% CI 0.08–0.17) in 2021(Fig. 4B), the burden distribution among countries with different SDIs is uneven.

### Burden of disease projections

**Fig. 5:**
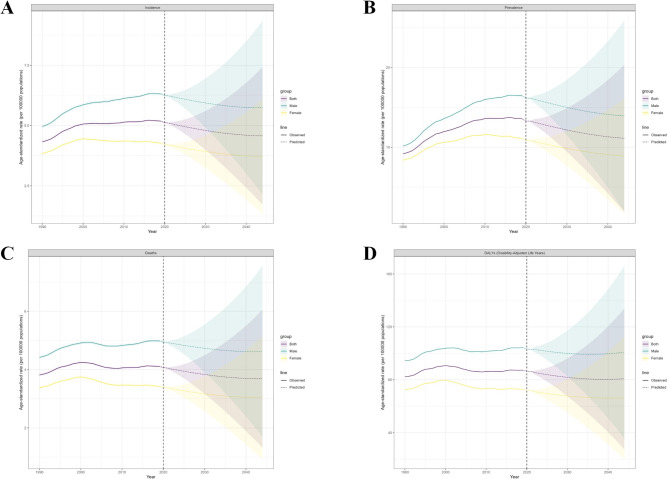
The ASIR (**A**), ASPR (**B**), ASMR (**C**) and ASDR (**D**) for global MM for the observational period (1990–2021) and the projection period (2022–2044).

Based on the GBD 2021 study, this study projects the global burden of disease for MM from 2022 to 2044. The results of the projections are shown in (Fig. 5). The global projections show that ASIR, ASPR, ASMR and ASDR show a decreasing trend by 2044 (Supplemental Table 4).

## Discussion

The 2021 GBD study, utilizing extensive data sources and advanced statistical modeling, offers the most comprehensive estimate of the burden of MM. To our knowledge, this is the first study to detail the incidence and rate of change of MM in adults over 40 from 1990 to 2021 at global, regional, and national levels. The data reveal a global increase in the incidence and prevalence of MM in this age group, consistent with previous studies that have reported similar trends in MM burden over the past decades. For instance, earlier research has also highlighted a steady rise in MM incidence globally, particularly in middle and low-middle SDI countries, due to improved diagnostic capabilities and aging populations^5^. However, our study uniquely identifies a more pronounced increase in middle, low-middle, and low SDI countries, a trend that has not been emphasized in prior analyses. This shift may reflect the combined effects of economic development, lifestyle changes, and enhanced healthcare access in these regions. The slower growth rates for DALYs and mortality align with other studies, which attribute this trend to treatment advancements like proteasome inhibitors and immunomodulatory drugs, significantly improving survival in high-SDI regions. However, our study also highlights persistent challenges in low-SDI regions, where access to these therapies remains limited, leading to higher mortality rates. This disparity underscores the need for targeted interventions in these areas, as noted in previous research^10^. The reduction in misdiagnosis rates, owing to improved screening tools, suggests that advancements in treatment may have enhanced patient survival and mitigated the overall disease burden. These trends reflect progress in MM management but also underscore the persistent public health challenge, especially in regions with lower SDI levels, where targeted interventions remain necessary. Globally, incidence rates of MM have been increasing in both males and females, consistent with previous epidemiological studies^4,11^; concurrent research has demonstrated that over the past 15 years, mortality rates among male patients have remained stable while those in female patients have shown a sustained decline, findings that suggest the gender-specific mortality disparity could lead to a widening disparity between male and female MM populations^6^.

With the increase of SDI, a steady rise in the incidence and prevalence of MM has been observed across most of the 21 regions, particularly in East Asia, including China. MM is notably common in provinces such as Shanghai, Zhejiang, and Beijing^12^. This trend can be attributed to several interrelated factors specific to MM. Rapid population aging in China has naturally led to a higher risk of MM, as the disease is more prevalent among older adults. Concurrently, significant lifestyle changes brought about by economic development, including Westernized dietary habits, reduced physical activity, and increased smoking rates, have been linked to elevated risks of MM^13^. Additionally, high levels of industrialization and economic development have resulted in significant environmental pollution, with long-term exposure to polluted air, water, and soil potentially contributing to the rising incidence of MM^14^. Advances in medical technology have also played a role, with improved diagnostic methods such as serum protein electrophoresis and bone marrow biopsies leading to more cases being detected, including at earlier stages. Furthermore, genetic predispositions in certain regions may exacerbate the impact of these environmental and lifestyle factors, resulting in higher MM incidence. By narrowing the focus to MM, these factors provide a clearer understanding of the disease’s epidemiology and its association with socio-economic development. In contrast, Low-SDI countries confront compounding systemic failures that perpetuate MM morbidity and mortality. Diagnostic capacities remain critically limited, with few Sub-Saharan African nations equipped to perform serum protein electrophoresis, leading to frequent misclassification of MM as chronic infections or nutritional disorders. Therapeutic disparities are profound, as novel agents like bortezomib remain virtually inaccessible, forcing reliance on corticosteroids and thalidomide as primary therapies. Workforce shortages exacerbate these challenges, with severe deficits in hematology specialists and fragmented referral pathways delaying specialist care by months. Competing public health priorities—particularly infectious disease control—and the absence of MM-specific policy frameworks further marginalize myeloma care, trapping patients in cycles of delayed diagnosis and suboptimal treatment.

Using data from the GBD 2021 study, this research projects the global burden of MM from 2022 to 2044. The forecast indicates a declining trend in the ASIR, ASPR, ASMR, and ASDR of MM, suggesting a reduced impact on individuals aged 40 and older in the coming decades. Nevertheless, effective management of MM-related health issues remains imperative, as MM continues to pose a significant public health challenge. Sustained efforts to mitigate high-risk factors, coupled with advancements in healthcare delivery, preventive strategies, and health promotion initiatives, are essential to further alleviate the global burden of this disease. It is noteworthy that the therapeutic landscape of MM has undergone a transformative evolution with the introduction of novel agents, leading to a substantial improvement in survival outcomes compared to historical benchmarks. During the era of conventional chemotherapy, the median survival duration for MM patients was merely 33 months. The advent and widespread adoption of autologous stem cell transplantation (ASCT), alongside the integration of novel therapeutic agents such as proteasome inhibitors (e.g., bortezomib, carfilzomib) and immunomodulatory drugs (e.g., thalidomide, lenalidomide, pomalidomide), have significantly extended patient survival. With the implementation of standardized treatment protocols, contemporary international data report a median survival of up to 126 months^15–17^. Looking forward, the clinical application of monoclonal antibodies, bispecific antibodies, and chimeric antigen receptor T-cell (CAR-T) therapies is anticipated to further enhance survival outcomes^18^.

This study faces several limitations. Firstly, the reliance on GBD 2021 data introduces potential inaccuracies, as some values are estimates rather than direct measurements. Due to gaps in disease surveillance across numerous countries, while GBD 2021 provides a structured framework for assessing the burden of MM, the potential for errors cannot be eliminated. Secondly, the study could not explore the impact of cancer stage or grading at diagnosis on mortality or morbidity due to limited data availability. Additionally, the effect of screening practices on the link between SDI and the burden of MM was not considered. Future research should employ a more comprehensive, population-based approach to validate these findings. Despite the advanced methodology and recognized reliability of the GBD database, concerns about data accuracy remain. In less developed regions, cancer cases may be underreported due to insufficient detection systems. Moreover, discrepancies in medical technology across different countries could lead to misdiagnoses or underdiagnoses of MM, thereby affecting the accuracy of the results.

## Conclusion

We conducted a comprehensive assessment of global, regional, and national trends in the incidence and burden of MM among adults over 40. MM represents an increasingly significant global health challenge, particularly in regions with low to middle SDI levels. Addressing MM in older populations should become a priority in global health policy discussions, especially in areas with lower SDI.

## Electronic supplementary material

Below is the link to the electronic supplementary material.

Supplementary Material 1

Supplementary Material 2

Supplementary Material 3

Supplementary Material 4

Supplementary Material 5

Supplementary Material 6

Supplementary Material 7

Supplementary Material 8

## Data Availability

All data generated or analysed during this study are included in this published article and its supplementary information files.

## Abbreviations

MM: Multiple myeloma
GBD: The global burden of disease
DALYs: Disability-adjusted life years
AAPC: The average annual percentage changes
APC: The annual percentage change
SDI: Socio-demographic index
ASIR: Age-standardized incidence rate
ASPR: Age-standardized prevalence rate
ASMR: Age-standardized mortality rate
ASDR: Age-standardized DALYs rate
UI: Uncertainty intervals
Cis: Confidence intervals
