# Supplementary material for: Trends and projections of the global and regional burden of multiple myeloma in adults aged 40 and over, 1990–2044
Source: Sci Rep. 2025 Apr 19;15:13595. doi: 10.1038/s41598-025-96981-w (PMC12009427; doi:10.1038/s41598-025-96981-w)
Supplement: Supplementary file 1 — Supplementary Material 1 [file 41598_2025_96981_MOESM1_ESM.docx]

| **China （1990-2021）** | **sex** | **Incidence** | **AAPC（95%UI）** | **Prevalence** | **AAPC（95%UI）** | **Deaths** | **AAPC（95%UI）** | **DALYs** | **AAPC（95%UI）** |
| --- | --- | --- | --- | --- | --- | --- | --- | --- | --- |
|  | **Both** |  | 4.66 (4.01-5.31) |  | 6.34 (5.75-6.94) |  | 3.89 (3.04-4.75) |  | 3.83 (3.29-4.37) |
|  | **Female** |  | 4.06 (3.39-4.73) |  | 5.53 (4.85-6.22) |  | 3.46 (2.69-4.24) |  | 3.29 (2.32-4.27) |
|  | **Male** |  | 5.11 (4.43-5.8) |  | 6.91 (6.42-7.4) |  | 4.18 (3.69-4.68) |  | 4.18 (3.67-4.7) |

Supplemental Table1: Time trends of MM incidence in China.
