# Supplementary material for: Trends and projections of the global and regional burden of multiple myeloma in adults aged 40 and over, 1990–2044
Source: Sci Rep. 2025 Apr 19;15:13595. doi: 10.1038/s41598-025-96981-w (PMC12009427; doi:10.1038/s41598-025-96981-w)
Supplement: Supplementary file 2 — Supplementary Material 2 [file 41598_2025_96981_MOESM2_ESM.docx]

Supplemental Table2: Decomposition analysis of change in incidence, Prevalence, mortality, and DALYs.

|  | **measure** | **Overll difference** | **Aging** | **Population** | **Epidemiological change** |
| --- | --- | --- | --- | --- | --- |
| **Global** |  |  |  |  |  |
|  | **Incidence** | 91521.25 | 9635.83 (10.53%) | 66853.45 (73.05%) | 15031.98 (16.42%) |
|  | **Prevalence** | 264691.93 | 16867.11 (6.37%) | 163707.25 (61.85%) | 84117.56 (31.78%) |
|  | **Deaths** | 67938.54 | 9454.63 (13.92%) | 54626.89 (80.41%) | 3857.02 (5.68%) |
|  | **DALYs (Disability-Adjusted Life Years)** | 1423955.51 | 124750.64 (8.76%) | 1224876.91 (86.02%) | 74327.95 (5.22%) |
| **High SDI** |  |  |  |  |  |
|  | **Incidence** | 34820.18 | 7617.72 (21.88%) | 23658.29 (67.94%) | 3544.16 (10.18%) |
|  | **Prevalence** | 118529.25 | 14301.79 (12.07%) | 61374.14 (51.78%) | 42853.32 (36.15%) |
|  | **Deaths** | 23279.44 | 7287.3 (31.3%) | 18864.64 (81.04%) | -2872.5 (-12.34%) |
|  | **DALYs (Disability-Adjusted Life Years)** | 366311.24 | 96162.96 (26.25%) | 379127.84 (103.5%) | -108979.56 (-29.75%) |
| **High-middle SDI** | |  |  |  |  |
|  | **Incidence** | 21839.24 | 2066.25 (9.46%) | 13265.3 (60.74%) | 6507.69 (29.8%) |
|  | **Prevalence** | 68351.05 | 3630.28 (5.31%) | 35660.17 (52.17%) | 29060.6 (42.52%) |
|  | **Deaths** | 15153.51 | 1986.56 (13.11%) | 10150.13 (66.98%) | 3016.82 (19.91%) |
|  | **DALYs (Disability-Adjusted Life Years)** | 319739.39 | 25615.35 (8.01%) | 237056.61 (74.14%) | 57067.44 (17.85%) |
| **Middle SDI** |  |  |  |  |  |
|  | **Incidence** | 22645.97 | 1569.05 (6.93%) | 11553.76 (51.02%) | 9523.16 (42.05%) |
|  | **Prevalence** | 55081.56 | 2447.19 (4.44%) | 25080.33 (45.53%) | 27554.04 (50.02%) |
|  | **Deaths** | 18206.88 | 1584.59 (8.7%) | 9903.06 (54.39%) | 6719.23 (36.9%) |
|  | **DALYs (Disability-Adjusted Life Years)** | 452534.5 | 24497.42 (5.41%) | 256137.16 (56.6%) | 171899.92 (37.99%) |
| **Low-middle SDI** | |  |  |  |  |
|  | **Incidence** | 9671.01 | 414.75 (4.29%) | 5550.11 (57.39%) | 3706.15 (38.32%) |
|  | **Prevalence** | 18152.05 | 447.79 (2.47%) | 9566.13 (52.7%) | 8138.12 (44.83%) |
|  | **Deaths** | 8902.53 | 474.63 (5.33%) | 5290.23 (59.42%) | 3137.68 (35.24%) |
|  | **DALYs (Disability-Adjusted Life Years)** | 223536.85 | 6578.9 (2.94%) | 137346.97 (61.44%) | 79610.98 (35.61%) |
| **Low SDI** |  |  |  |  |  |
|  | **Incidence** | 2441.27 | -93.69 (-3.84%) | 1868.75 (76.55%) | 666.21 (27.29%) |
|  | **Prevalence** | 4256.61 | -163.74 (-3.85%) | 2969.58 (69.76%) | 1450.77 (34.08%) |
|  | **Deaths** | 2322.94 | -83.56 (-3.6%) | 1832.32 (78.88%) | 574.18 (24.72%) |
|  | **DALYs (Disability-Adjusted Life Years)** | 60347.67 | -2538.51 (-4.21%) | 48616.22 (80.56%) | 14269.97 (23.65%) |
