# Supplementary material for: Trends and projections of the global and regional burden of multiple myeloma in adults aged 40 and over, 1990–2044
Source: Sci Rep. 2025 Apr 19;15:13595. doi: 10.1038/s41598-025-96981-w (PMC12009427; doi:10.1038/s41598-025-96981-w)
Supplement: Supplementary file 3 — Supplementary Material 3 [file 41598_2025_96981_MOESM3_ESM.docx]

Supplemental Table3: Slope indices and Concentration indices inequality for ASDR of MM in 1990 and 2021.

|  | **Slope (95% CI)** | **Concentration index (95% CI)** |
| --- | --- | --- |
| **Incidence** |  |  |
| **1990** | 6.37  (7.65-5.09) | 0.4  (0.33-0.47) |
| **2021** | 8.22  (9.57-6.87) | 0.22  (0.16-0.27) |
| **Prevalence** |  |  |
| **1990** | 14.02  (16.78-11.27) | 0.51  (0.42-0.6) |
| **2021** | 25.16  (29.36-20.97) | 0.36  (0.28-0.44) |
| **Deaths** |  |  |
| **1990** | 5.07  (6.1-4.04) | 0.35  (0.29-0.41) |
| **2021** | 5.24  (6.14-4.34) | 0.14  (0.1-0.19) |
| **DALYs** |  |  |
| **1990** | 113.19  (136.08-90.29) | 0.33  (0.27-0.39) |
| **2021** | 110.76  (130.77-90.75) | 0.12  (0.08-0.17) |
