# Supplementary material for: Trends and projections of the global and regional burden of multiple myeloma in adults aged 40 and over, 1990–2044
Source: Sci Rep. 2025 Apr 19;15:13595. doi: 10.1038/s41598-025-96981-w (PMC12009427; doi:10.1038/s41598-025-96981-w)
Supplement: Supplementary file 4 — Supplementary Material 4 [file 41598_2025_96981_MOESM4_ESM.docx]

Supplemental Table4: The number of cases of prediction of incidence, prevalence, deaths and DALYs of nasopharyngeal carcinoma from 2022–2044

| Global |  |  | 2022 | 2044 |
| --- | --- | --- | --- | --- |
|  | Incidence | Male | 6.2(6.07-6.32) | 5.75 (2.14-9.36) |
|  |  | Female | 4.16(4.07-4.24) | 3.72 (1.34-6.1) |
|  |  | Both | 5.06(4.97-5.16) | 4.58 (1.72-7.43) |
|  | Prevalence | Male | 16.03(15.58-16.47) | 13.94 (2-25.87) |
|  |  | Female | 10.73(10.41-11.06) | 8.88 (1.65-16.11) |
|  |  | Both | 13.15(12.77-13.54) | 11.12 (1.91-20.32) |
|  | Deaths | Male | 4.9(4.8-5) | 4.63 (1.7-7.55) |
|  |  | Female | 3.34(3.27-3.41) | 3.04 (0.93-5.15) |
|  |  | Both | 4.02(3.95-4.09) | 3.69 (1.32-6.06) |
|  | DALYs | Male | 102.9(100.79-105.01) | 100.93 (35.76-166.09) |
|  |  | Female | 71.01(69.7-72.33) | 66.30 (19.96-112.64) |
|  |  | Both | 85.46(83.96-86.97) | 80.80 (27.59-134.01) |
