# Supplementary material for: Trends and projections of the global and regional burden of multiple myeloma in adults aged 40 and over, 1990–2044
Source: Sci Rep. 2025 Apr 19;15:13595. doi: 10.1038/s41598-025-96981-w (PMC12009427; doi:10.1038/s41598-025-96981-w)
Supplement: Supplementary file 5 — Supplementary Material 5 [file 41598_2025_96981_MOESM5_ESM.docx]

Supplementary Materials: Detailed Descriptions of GBD Metrics

This document provides detailed descriptions of standard Global Burden of Disease (GBD) metrics, including Disability-Adjusted Life Years (DALYs), Average Annual Percentage Change (AAPC), and Socio-Demographic Index (SDI). These descriptions have been moved here to improve the readability of the main manuscript.

DALYs, ASIR, ASPR, ASMR and ASDR

DALYs are computed by adding the years of life lost (YLL) due to early death to the years lived with disability (YLD). Each DALY reflects the loss of one year of healthy life, which includes both the years spent living with a disability and those lost due to death. To adjust for variations in age distributions across different regions, various age-standardized metrics were employed. These include the age-standardized incidence rate (ASIR, per 100,000 per year), the age-standardized prevalence rate (ASPR, per 100,000 per year), the age-standardized mortality rate (ASMR, per 100,000 per year), and the age-standardized DALYs rate (ASDR, per 100,000 per year).

APC and AAPC

To determine age-specific rates and their AAPC, linear regression was applied with rates on a logarithmic scale as the dependent variable and year as the independent variable. The APC was calculated by geometrically averaging the yearly percentage changes obtained from the regression analysis. The AAPC, representing a weighted average of the APCs, summarizes the average rate changes over several years into a single figure. The AAPC values, along with their 95% confidence intervals (CIs), illustrate the general trends in the rates, indicating whether the rates are increasing, decreasing, or stable annually. For example, an AAPC of 0.5 would signify a 0.5% yearly increase in the rate.

SDI

The SDI is a composite measure that integrates a country's per capita income, adjusted for historical lag, the average years of schooling, and the fertility rate among women under 25. Each component is standardized using specific health metrics, resulting in values ranging from 0 to 1, where higher scores reflect more advanced socio-economic development. In the GBD 2021 study, countries and territories were classified into five SDI tiers: low, low-middle, middle, high-middle, and high.[1]

1. *Global incidence, prevalence, years lived with disability (YLDs), disability-adjusted life-years (DALYs), and healthy life expectancy (HALE) for 371 diseases and injuries in 204 countries and territories and 811 subnational locations, 1990-2021: a systematic analysis for the Global Burden of Disease Study 2021.* Lancet, 2024. 403(10440): p. 2133-2161.
