# Supplementary figures and images for: Trends and projections of the global and regional burden of multiple myeloma in adults aged 40 and over, 1990–2044
Source: Sci Rep. 2025 Apr 19;15:13595. doi: 10.1038/s41598-025-96981-w (PMC12009427; doi:10.1038/s41598-025-96981-w)

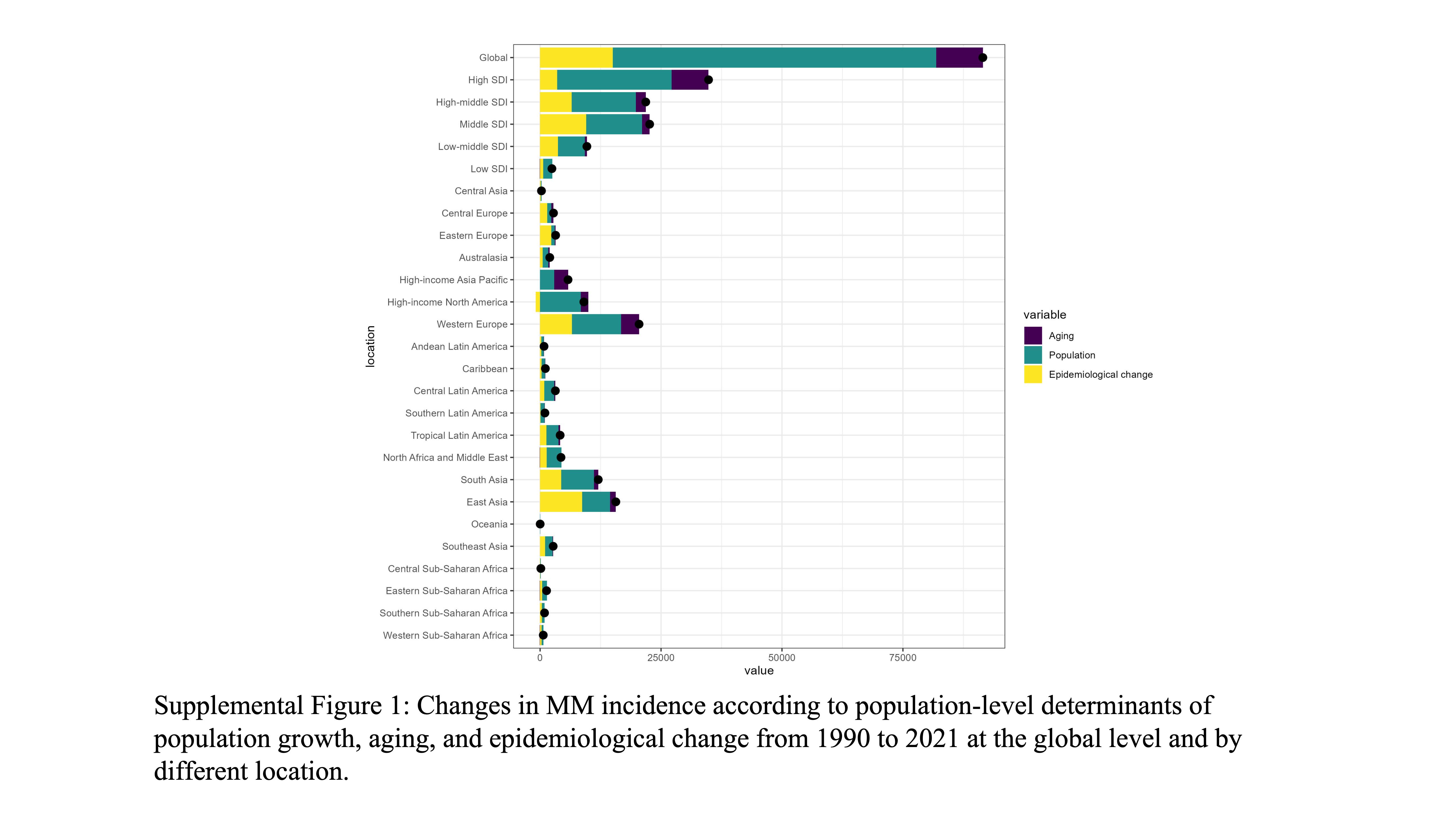

Supplement: Supplementary file 6 — Supplementary Material 6 [file 41598_2025_96981_MOESM6_ESM.jpg]

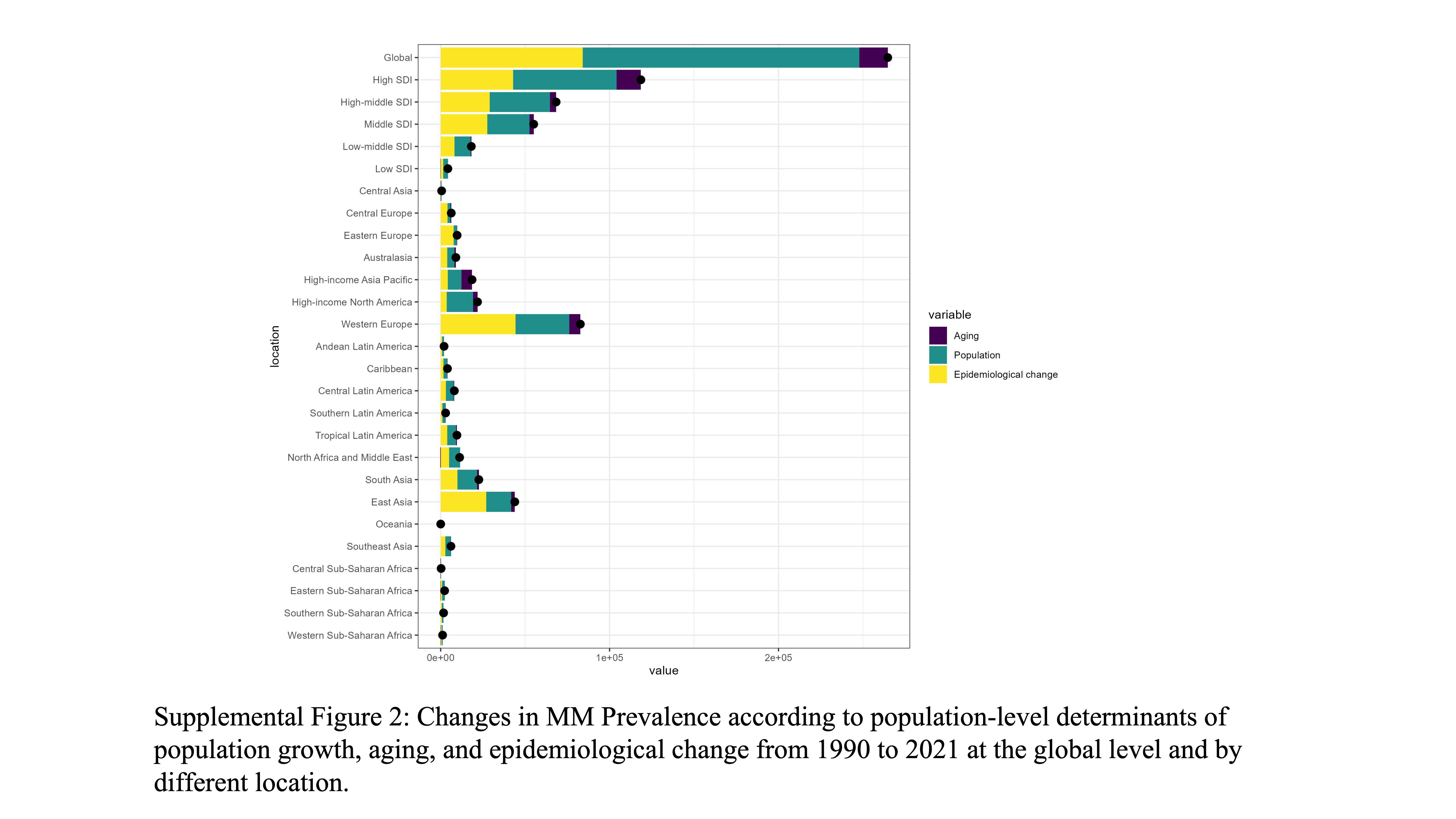

Supplement: Supplementary file 7 — Supplementary Material 7 [file 41598_2025_96981_MOESM7_ESM.jpg]

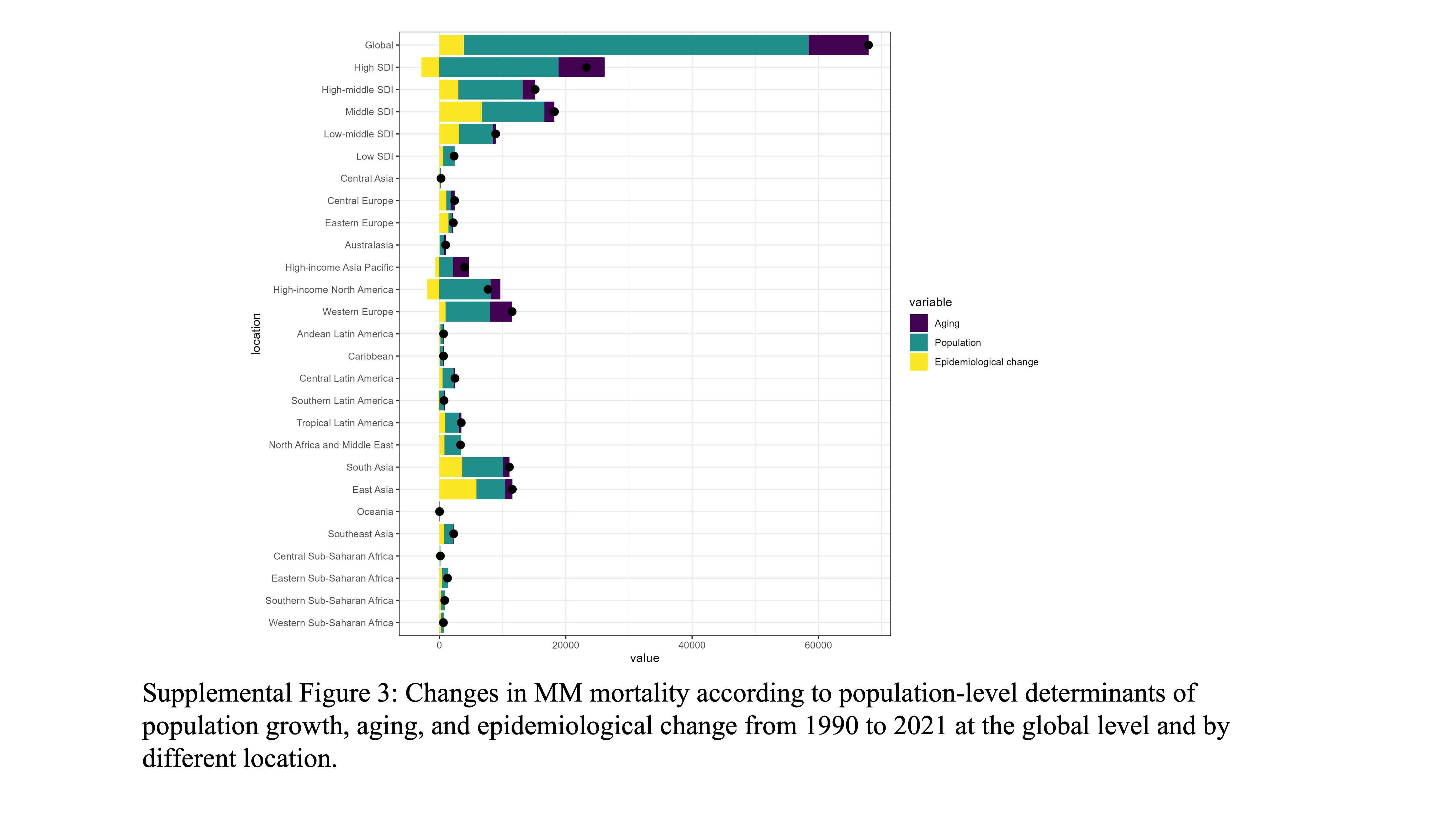

Supplement: Supplementary file 8 — Supplementary Material 8 [file 41598_2025_96981_MOESM8_ESM.jpg]
